# Risk of major mental disorders in the offspring of parents with migraine

**DOI:** 10.1186/s12991-024-00508-y

**Published:** 2024-06-22

**Authors:** Dian-Jeng Li, Shih-Jen Tsai, Tzeng-Ji Chen, Chih-Sung Liang, Mu-Hong Chen

**Affiliations:** 1 Department of Addiction Science, Kaohsiung Municipal Kai-Syuan Psychiatric Hospital, Kaohsiung, Taiwan; 2 Department of Nursing, Meiho University, Pingtung, Taiwan; 3 Department of Psychiatry, Taipei Veterans General Hospital, Taipei, Taiwan; 4 Department of Psychiatry, College of Medicine, National Yang Ming Chiao Tung University, No. 201, Sec. 2, Shihpai Road, Beitou District, Taipei, 11217 Taiwan; 5 Department of Family Medicine, Taipei Veterans General Hospital, Taipei, Taiwan; 6 Institute of Hospital and Health Care Administration, National Yang Ming Chiao Tung University, Taipei, Taiwan; 7 Department of Family Medicine, Hsinchu Branch, Taipei Veterans General Hospital, Hsinchu, Taiwan; 8 Department of Psychiatry, Beitou Branch, Tri-Service General Hospital, National Defense Medical School, Taipei, Taiwan; 9 Department of Psychiatry, National Defense Medical School, No. 60, Xinmin Road, Beitou District, Taipei, 11243 Taiwan

**Keywords:** Bipolar disorder, Depressive disorder, Child and adolescence psychiatry, Migraine

## Abstract

**Background:**

Migraine has been associated with mental disorders, however whether parental migraine is associated with an increased risk of major mental disorders (MMDs) in offspring has not been investigated. We aimed to examine the risk of the development of MMDs in the offspring of parents with migraine compared with those of parents without migraine.

**Methods:**

This study used data derived from the Taiwan National Health Insurance Research Database. Offspring of parents with migraine and a control group consisting of offspring of parents without migraine matched for demographic and parental mental disorders were included. Cox regression was used to estimate the risk of MMDs, including schizophrenia, depressive disorder, bipolar disorder, autistic spectrum disorder (ASD), and attention deficit/hyperactivity disorder (ADHD). Sub-analyses stratified by the fathers and mothers were further performed to separately clarify the risks of MMDs among the offspring.

**Results:**

We included 22,747 offspring of parents with migraine and 227,470 offspring of parents without migraine as the controls. Parental migraine was significantly associated with an increased risk of ADHD (reported as hazard ratios with 95% confidence intervals: 1.37, 1.25–1.50), bipolar disorder (1.35, 1.06–1.71), and depressive disorder (1.33, 1.21–1.47) compared to the offspring of parents without migraine. Importantly, sub-analyses showed that only maternal migraine was significantly associated with these risks.

**Conclusions:**

Due to the heavy burden of MMDs, healthcare workers should be aware of the risk of MMDs in the offspring of parents with migraine, particular in mothers.

**Supplementary Information:**

The online version contains supplementary material available at 10.1186/s12991-024-00508-y.

## Introduction

### Characteristics of migraine

Migraine is a common headache disorder caused by increased excitability of the central nervous system, and it is a major cause of disability [1]. Migraine and other headaches account for about 3% of all emergency department visits annually in the US and they are the fourth leading reason for hospital visits [2]. Migraine is characterized as a unilateral pulsating headache accompanied with aural symptoms including nausea, vomiting, photophobia, and phonophobia [3]. The prevalence of migraine varies among races, with reported rates of 15.4% in non-Hispanic White, 10.7% in Asian, and 15.7% in Black populations [4]. One in six individuals in the US are affected by migraines [2]. Importantly, migraine is associated with multiple burdens which can affect the quality of life and social function [5, 6]. For example, a previous study reported that migraine was associated with heavy disability, economic impact, psychiatric comorbidities, and health care resource utilization [7]. Due to this heavy burden associated with migraine, further research regarding the comorbidities may be beneficial to better understand the etiologies.

### Associations between migraine and mental illness

Previous studies have investigated the association between migraine and mental health problems [7], including depression, anxiety, bipolar disorder, posttraumatic stress disorder, personality disorders, and even suicide attempts [8]. A population-based cohort study in Canada reported that depressive disorder, bipolar disorder, social phobia, and anxiety-related disorders were at least twice as prevalent in people with migraine compared with controls after adjusting for demographic and socioeconomic variables [9]. Moreover, previous studies have reported a bidirectional relationship between migraine and sleep disorder [10, 11]. In addition to common adult mental disorders, a positive association between attention deficit/hyperactivity disorder (ADHD) and migraine has also been reported [12, 13]. Several possible theories have been proposed to explain the association between migraine and mental disorders, including shared biological factors [14]. Evidence suggests an association between the dopamine D_2_ receptor genotype with migraine, depressive disorder, generalized anxiety disorder, phobia, and panic attacks [15]. The association between ADHD and migraine has been associated with dopaminergic dysfunction, GABAergic dysfunction, and shared genetic factors [16, 17]. In addition, serotonin receptors, serotonin transporters, and catecholamines have also been implicated in migraine and various mental disorders [18, 19]. Considering the complicated etiologies and poor prognosis of patients with migraine and mental illness [20, 21], clinicians should be aware about this comorbidity.

### Aim of the current study

Since previous research has demonstrated genetic effects on migraine and mental illness [22, 23], we were interested in exploring the association between migraine in parents and the subsequent development of major mental disorders (MMDs) in their offspring. Considering the heavy burden caused by migraine and mental illness, exploring this association between parental migraine and MMDs among offspring for the early detection and interventions for possible subsequent MMDs is an important issue. A nationwide registration study reported that parental migraine, even in the absence of parental bipolar disorder, was a risk factor for the development of bipolar disorder among offspring [24]. However, no further studies have investigated the association between parental migraine and the risk of MMDs in their offspring, or whether there are differences in such an association between fathers and mothers. Given this knowledge gap, we aimed to investigate the association between parental migraine and the risk of MMDs among their offspring using a population-based cohort study. We hypothesized that parental migraine may be associated with the risk of the subsequent development of MMDs among their offspring, and that this risk may differ between paternal and maternal migraine.

## Methods

### Data source

The Taiwan National Health Research Institute audits and releases the Taiwan National Health Insurance Research Database (NHIRD) for scientific studies [25, 26]. The Taiwan NHIRD comprises comprehensive healthcare data of > 99.7% of the population in Taiwan, including demographic data, clinical visit dates, and disease diagnoses. All insurance claims information is anonymous to maintain privacy. The diagnostic codes used are based on the International Classification of Diseases, 9th Revision, Clinical Modification (ICD-9-CM). The NHIRD has been used extensively in many Taiwanese epidemiologic studies [27–30]. Using Tsai et al.’s and Cheng et al.’s methods, we constructed the familial pedigree, including the parent-child relationship [31, 32]. Only those who can be clearly linked to their parents (fathers and mothers) were included in the analysis of the present study. The study protocol was reviewed and accepted by the Institutional Review Board of Taipei Veterans General Hospital (approval number: TPEVGH-IRB-2018-07-016AC).

### Inclusion criteria for the offspring of parents with migraine

**Fig. 1:**
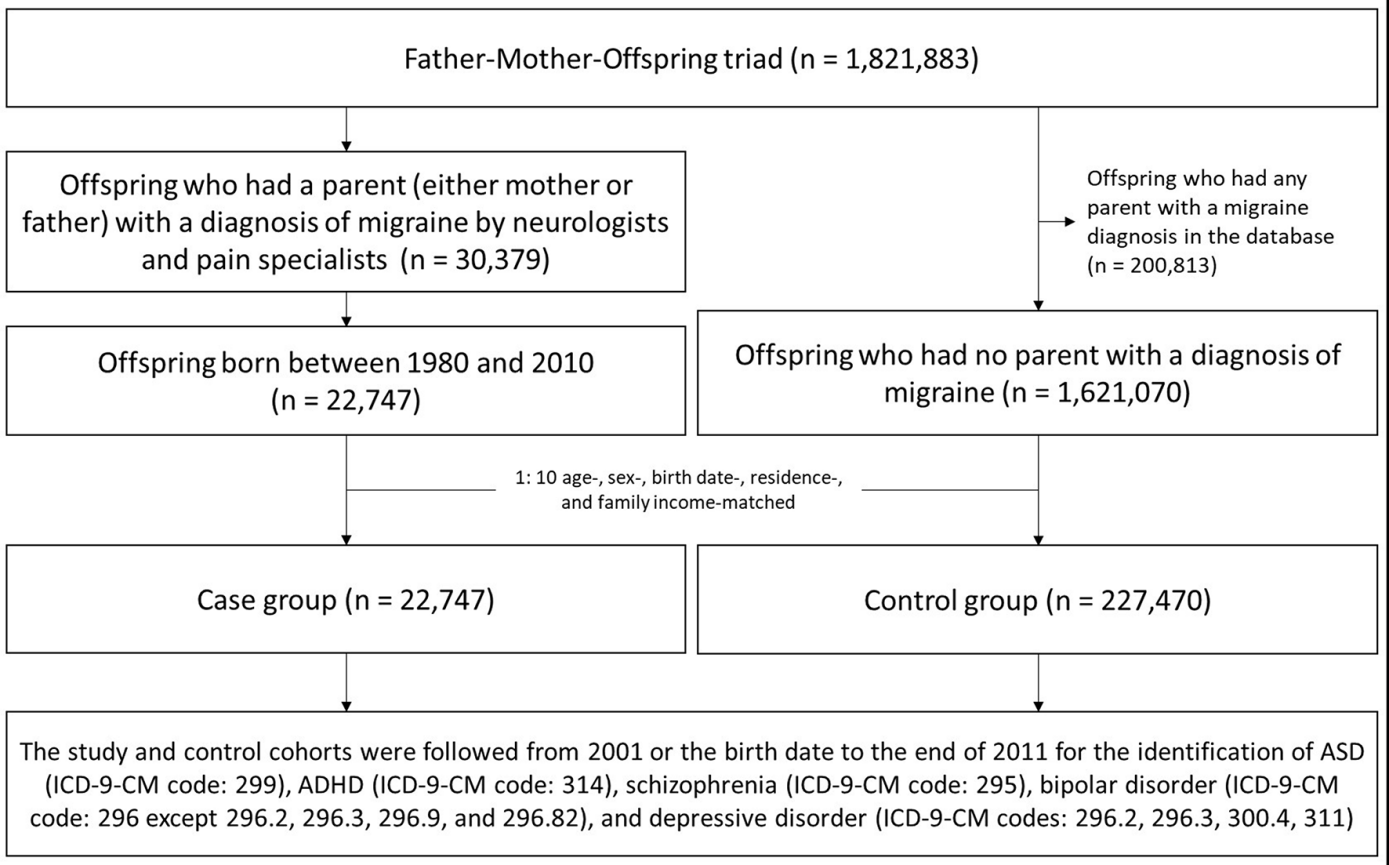
Flow chart of the study design

Individuals born between 1980 and 2010 who had a parent (either mother or father) with a diagnosis of migraine (ICD-9-CM code: 346) given at least twice by board-certified neurologists and pain specialists were enrolled as the study cohort. A 1:10 age-, sex-, birth date-, residence-, and family income-matched control cohort was randomly identified after eliminating the study cases and those who had parents with migraine anytime in the database. The study and control cohorts were followed from 2001 or the birth date to the end of 2011, and the occurrence of MMDs including autistic spectrum disorder (ASD; ICD-9-CM code: 299), ADHD (ICD-9-CM code: 314), schizophrenia (ICD-9-CM code: 295), bipolar disorder (ICD-9-CM code: 296 except 296.2, 296.3, 296.9, and 296.82), and depressive disorder (ICD-9-CM codes: 296.2, 296.3, 300.4, 311) was recorded. We only selected the first (main) diagnosis of mental disorder as identification of MMDs if patients had multiple comorbidities of mental disorders. Moreover, the initial diagnosis of MMDs would be identified as index diagnosis of MMDs, and the other diagnoses of mental disorder occurred in the future would not be analyzed during the study period. Parental schizophrenia, bipolar disorder, and depressive disorder were also assessed between 2000 and 2011 as confounding factors. The aforementioned MMDs were diagnosed at least twice by board-certified psychiatrists to improve the diagnostic validity. The level of urbanization (level 1 to level 5; level 1: most urbanized region; level 5: least urbanized region) was also recorded [33]. Family income and urbanization were defined by the most recent recording data in the database. The flow chart of study design was listed in the Fig. 1.

### Statistical analysis

For between-group comparisons, the F test was used for continuous variables and Pearson’s χ^2^ test for nominal variables, where appropriate. Cox regression analyses with full adjustment of demographic data (age, sex, residence, and income) and parental mental disorders were used to calculate the hazard ratios (HRs) with 95% confidence intervals (CIs) of subsequent MMDs, including ASD, ADHD, schizophrenia, bipolar disorder, and depressive disorder, in the offspring of the parents with migraine compared with those of the parents without migraine. Sub-analyses stratified by fathers and mothers were further performed to separately clarify the risks of subsequent MMDs in the offspring of the fathers and mothers with migraine. The reference group of are offspring of fathers without migraine and offspring of mothers without migraine.

A 2-tailed *P*-value of less than 0.05 was considered statistically significant. All data processing and statistical analyses were performed using SPSS version 17 (IBM Inc., Armonk, NY) and SAS version 9.1 (SAS Institute, Cary, NC).

## Results

### Demographic and clinical characteristics

A total of 22,747 offspring of parents with migraine and 227,470 offspring of parents without migraine were included, with mean ages of 8.88 (standard deviation; SD: 6.97) and 8.89 (6.98) years, respectively. The parents with migraine had higher rates of mental comorbidities including schizophrenia (1.1% vs. 0.7%, *p* < 0.001), bipolar disorder (1.8% vs. 0.7%, *p* < 0.001), and depressive disorder (7.1% vs. 2.6%, *p* < 0.001) than the parents without migraine. Regarding the incidence of MMDs, the offspring of parents with migraine had a higher incidence of ADHD (2.4% vs. 1.7%, *p* < 0.001), bipolar disorder (0.4% vs. 0.2%, *p* < 0.001), and depressive disorder (2.0% vs. 1.4%, *p* < 0.001) than the control group. Other details are shown in Table 1.

**Table 1:** Demographic characteristics and incidence of major mental disorders between the offspring of parents with or without migraine

	Offspring of parents with migraine (*n* = 22,747)	Offspring of parents without migraine (*n* = 227,470)	*p*-value
Age (years, SD)	8.88 (6.97)	8.89 (6.98)	0.851
Birth-year (n, %)			0.998
1980–1984	5041 (22.2)	50,415 (22.2)	
1985–1989	41.61 (18.2)	41,758 (18.4)	
1990–1994	4358 (19.2)	43,506 (19.1)	
1995–1999	4138 (18.2)	41,174 (18.1)	
≥ 2000	5053 (22.2)	50,617 (22.2)	
Sex (n, %)			1.000
Male	11,867 (52.2)	118,670 (52.2)	
Female	10,880 (47.8)	108,800 (47.8)	
Parents with migraine (n, %)			
Fathers	6379 (28.0)		
Mothers	16,387 (72.0)		
Parental mental comorbidities (n, %)			
Schizophrenia	247 (1.1)	1564 (0.7)	< 0.001
Bipolar disorder	415 (1.8)	1611 (0.7)	< 0.001
Depressive disorder	1605 (7.1)	5868 (2.6)	< 0.001
Incidence of major mental disorder (n, %)			
ASD	71 (0.3)	651 (0.3)	0.479
Age at diagnosis (years, SD)	9.51 (5.78)	8.40 (5.56)	0.113
ADHD	541 (2.4)	3798 (1.7)	< 0.001
Age at diagnosis (years, SD)	9.09 (3.63)	8.92 (3.76)	0.339
Schizophrenia	71 (0.3)	653 (0.3)	0.481
Age at diagnosis (years, SD)	19.86 (3.58)	20.40 (4.11)	0.288
Bipolar disorder	81 (0.4)	531 (0.2)	< 0.001
Age at diagnosis (years, SD)	22.09 (5.22)	21.32 (4.23)	0.158
Depressive disorder	456 (2.0)	3189 (1.4)	< 0.001
Age at diagnosis (years, SD)	20.81 (4.66)	21.16 (4.33)	0.108
Level of urbanization (n, %)			1.000
1 (most urbanized)	4722 (20.8)	47,220 (20.8)	
2	7116 (31.3)	71,160 (31.3)	
3	2842 (12.5)	28,420 (12.5)	
4	2350 (10.3)	23,500 (10.3)	
5 (most rural)	5717 (25.1)	57,170 (25.1)	
Income-related insured amount (n, %)			1.000
≤ 19,100 NTD/month	2950 (13.0)	29,500 (13.0)	
19,001 ~ 42,000 NTD/month	7635 (33.6)	76,350 (33.6)	
> 42,000 NTD/month	12,162 (53.4)	121,620 (53.4)	

SD: standard deviation; NTD: new Taiwan dollars; ASD: autism spectrum disorder; ADHD: attention deficit hyperactivity disorder

### Risk of MMDs among the offspring of parents with migraine compared with the controls

**Fig. 2:**
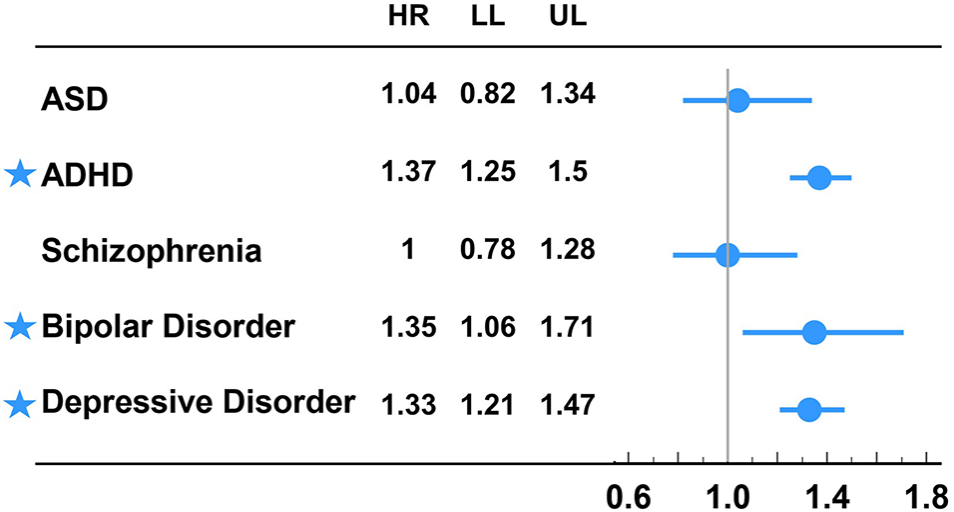
Risks of subsequent major mental disorders between the offspring of parents with or without migraine†. ADHD: attention deficit hyperactivity disorder; ASD: autism spectrum disorder; HR: hazard ratio; LL: lower limits of 95% confidence interval; UL: upper limits of 95% confidence interval. †: adjusting for demographic data and parental mental comorbidities

After adjusting for age, sex, residence, income, and parental mental comorbidities, the offspring of parents with migraine had a higher risk of ADHD (reported as HR with 95% Cl: 1.37, 1.25–1.50), bipolar disorder (1.35, 1.06–1.71), and depressive disorder (1.33, 1.21–1.47) compared to the control group (Supplementary Table 1). We did not find significant risks for ASD and schizophrenia. In addition, we found that the offspring of mothers with migraine had a higher risk of ADHD (1.43, 1.29–1.59), bipolar disorder (1.41, 1.07–1.87), and depressive disorder (1.46, 1.31–1.63) compared with the control group (Table 2), but again no significant risks were found for ASD or schizophrenia. We also found that the offspring of fathers with migraine did not have significantly higher risks of any MMD compared with the control group (Table 2). These findings are visualized in Fig. 2. In our study, the HRs that were statistically significant between 1.33 and 1.46. Therefore, the log HRs ranged between 0.12 and 0.16, and the values may not be easily understandable. So, we still used the HRs on an arithmetic scale in forest plot.

**Table 2:** Risks of subsequent major mental disorders between the offspring of parents with or without migraine, stratified by fathers and mothers †

	ASD	ADHD	Schizophrenia	Bipolar disorder	Depressive disorder
	**All offspring sample**				
Offspring of fathers without migraine (reference, n, %)	181 (0.3)	1113 (1.7)	176 (0.3)	158 (0.2)	844 (1.3)
Offspring of fathers with migraine (n, %)	22 (0.3)	134 (2.1)	19 (0.3)	22 (0.3)	83 (1.3)
HR (95% CI)	1.20 (0.77–1.87)	1.18 (0.99–1.42)	1.00 (0.62–1.61)	1.22 (0.78–1.92)	0.94 (0.75–1.18)
Offspring of mothers without migraine (reference, n, %)	470 (0.3)	2689 (1.6)	477 (0.3)	374 (0.2)	2347 (1.4)
Offspring of mothers with migraine (n, %)	49 (0.3)	408 (2.5)	52 (0.3)	59 (0.4)	373 (2.3)
HR (95% CI)	1.00 (0.74–1.34)	**1.43 (1.29–1.59)**	1.01 (0.75–1.34)	**1.41 (1.07–1.87)**	**1.46 (1.31–1.63)**
	**Male offspring sample**				
Offspring of fathers without migraine (reference, n, %)	152 (0.5)	904 (3.7)	92 (0.3)	80 (0.2)	376 (1.1)
Offspring of fathers with migraine (n, %)	18 (0.5)	105 (3.1)	12 (0.4)	13 (0.4)	35 (1.0)
HR (95% CI)	1.15 (0.70–1.87)	1.14 (0.93–1.40)	1.21 (0.66–2.21)	1.47 (0.81–2.65)	0.86 (0.61–1.22)
Offspring of mothers without migraine (reference, n, %)	394 (0.5)	2149 (2.5)	253 (0.3)	170 (0.2)	968 (1.1)
Offspring of mothers with migraine (n, %)	41 (0.5)	316 (3.7)	27 (0.3)	21 (0.2)	162 (1.9)
HR (95% CI)	0.99 (0.71–1.36)	**1.40 (1.25–1.58)**	0.99 (0.67–1.48)	1.05 (0.66–1.66)	**1.56 (1.32–1.85)**
	**Female offspring sample**				
Offspring of fathers without migraine (reference, n, %)	29 (0.1)	209 (0.7)	84 (0.3)	78 (0.3)	468 (1.6)
Offspring of fathers with migraine (n, %)	4 (0.1)	29 (1.0)	7 (0.2)	9 (0.3)	48 (1.6)
HR (95% CI)	1.30 (0.46–3.72)	1.33 (0.90–1.97)	0.82 (0.38–1.78)	1.05 (0.53–2.11)	1.00 (0.74–1.35)
Offspring of mothers without migraine (reference, n, %)	76 (0.1)	540 (0.7)	224 (0.3)	204 (0.3)	1379 (1.8)
Offspring of mothers with migraine (n, %)	8 (0.1)	92 (1.2)	25 (0.3)	38 (0.5)	211 (2.7)
HR (95% CI)	1.03 (0.50–2.14)	**1.61 (1.29–2.01)**	1.00 (0.66–1.52)	**1.68 (1.19–2.39)**	**1.40 (1.21–1.62)**

HR: hazard ratio; CI: confidence interval; ASD: autism spectrum disorder; ADHD: attention deficit hyperactivity disorder

†: adjusting for demographic data and parental mental comorbidities

**Bold** indicates statistical significance

## Discussion

### Main findings of the current study

In this study, we investigated the association between parental migraine and subsequent MMDs among their offspring. Compared with the offspring of parents without migraine, the risks of subsequent ADHD, bipolar disorder, and depressive disorder were increased in the offspring of parents with migraine. Moreover, these risks were different between the mothers and fathers. We observed that only the offspring of mothers with migraine were associated with significant risks of ADHD, bipolar disorder, and depressive disorder, but not those without migraine. These findings show the unique role of mothers with migraine on the subsequent development of ADHD, bipolar disorder, and depressive disorder in their offspring.

### Parental migraine and the occurrence of MMDs among their offspring

Evidence has suggested an association between migraine and mental disorders, and our study further extends this association to a trans-generational effect. Several reasons may explain our findings. First, genetic and heredity factors may contribute to the subsequent development of MMDs among offspring. Some shared susceptibility genes may play a role in familial clustering and elevated comorbidity of migraine and MMDs. For instance, previous genetic studies have indicated a genetic overlap between bipolar disorder and migraine [34, 35]. Therefore, genetic factors may be involved in the trans-generational association of parental migraine with MMDs among their offspring. In addition, twin studies have estimated the heritability of migraine to be about 45%, indicating that genetic factors play a substantial role in the familial transmission of migraine [36]. Another genome-wide association study identified 38 risk loci for migraine [37]. The heredity of migraine from parents to their offspring may also be associated with the subsequent development of MMDs among their offspring, considering the aforementioned association between migraine and MMDs.

Second, environmental factors may also have contributed to our findings. Previous studies have discussed the association between parental migraine and childhood trauma, maltreatment, or impaired wellbeing, and that parental migraine has a negative impact on children, particularly in the domains of global wellbeing and parent/child relationship [38]. Other studies have also reported a great impact on home/family life [39] and the relationship between the parent with migraine and his/her children [40]. Recently, an analysis of data derived from the Chronic Migraine Epidemiology and Outcomes Study showed the impacts of parental migraine on children and adolescents [41]. In that study, adolescents reported that parental migraine disturbed support from their parents, their emotional experiences, school life, and group activities. In addition, mothers with migraine have reported that their children had lower self-esteem and poorer social relationships than the children of mothers without migraine [42], indicating the predominantly psychological burden among children. Furthermore, childhood trauma or maltreatment may give rise to the development of mental health problems. Previous studies have reported an association between childhood trauma or parental maltreatment with an elevated risk of psychosis and mental disorders [43, 44]. With a gene-environment approach, another meta-analysis also demonstrated a potential causal role of childhood maltreatment on depression, schizophrenia and ADHD [45]. Taken together, parental migraine may be associated with childhood maltreatment or a negative impact on the mental health of their offspring, leading to the development of MMDs. On the other hand, a previous study demonstrated that parental depression, antisocial behavior and drug dependence were associated with offspring migraine [46]. This echoes the findings of the current study and highlight the intertwined relationship between migraine and mental illness across generations.

### The association between maternal migraine and MMDs in their offspring

Another interesting finding in this study is that only maternal migraine played a predominant role in the subsequent development of MMDs among their offspring. The mother-specific transmission of migraine to their offspring may be a possible cause. A large, population-based cohort study from Norway demonstrated that parental migraine was associated with offspring migraine, with a stronger association for maternal migraine [47]. Another study showed that female sex was a significant risk factor for migraine, and a sex-biased transmission was also observed [48]. This mother-specific transmission of migraine may explain the development of MMDs in their offspring. On the other hand, the unique role of the mother in parenting style may be another factor. A previous study suggested that mothers of Han-Chinese families take the main parenting role, and that they are more supportive and responsive than fathers [49]. Accordingly, we hypothesize that maternal migraine may have a great impact on childhood maltreatment, leading to the development of MMDs. However, further studies are warranted to explore the detailed etiology of maternal migraine and the development of MMDs among their offspring.

### Strengths and limitations

We used the NHIRD for the analysis, which had the strengths of coverage and generalizability for Taiwanese’s population. The Taiwan National Health Insurance was established in 1995 to deliver universal coverage for the entire population of Taiwan and is also a compulsory nationwide health insurance system. Every subject in the NHIRD can be followed from the insurance start to the death. This insurance program covered 99.0% of the population by 2004 and more than 99.9% by 2014, and includes almost all medical visits and hospitalizations [50, 51]. Therefore, missing data or detection bias of Taiwanese’s population can be minimal. The current study has several limitations. First, we did not include individuals who did not seek medical care for migraines. These subjects may have taken over-the-counter drugs to relieve their pain, and as such they would not have been recorded in the NHIRD. Second, as the NHIRD is an observational database, it is possible that residual confounding factors remain. However, this naturalistic observation design may demonstrate clinical practice in the real world. Due to the limitation of database, we defined family income and residence based on the most recent recording data. However, family income and residence may change over time. Finally, the risk of the offspring of two parents with migraine was unavailable due to relatively small sample size (*n* = 19).

## Conclusion

In this study, we found a predominant association between parental migraine and the risk of MMDs among their offspring. Moreover, maternal migraine had a greater impact on the development of MMDs among their offspring then paternal migraine. Our study highlights the importance of early identification and intervention for parents with migraine, especially mothers, to prevent the risk of the subsequent development of MMDs among their offspring. Healthcare workers should be aware of parents with migraine due to the possible risk of parenting dysfunction, which may lead to an increased risk of MMDs among their children. On the other hand, the genetic susceptibility in the association between parental migraine and subsequent development of MMDs among offspring also highlight the importance of early detection and psychoeducation for those parents who have migraine. Further neuro-biological studies are necessary to explore the etiology of the association between parental migraine and MMDs among their offspring.

### Electronic supplementary material

Below is the link to the electronic supplementary material.

Supplementary Material 1

## Data Availability

No datasets were generated or analysed during the current study.
