# Supplementary material for: Risk of major mental disorders in the offspring of parents with migraine
Source: Ann Gen Psychiatry. 2024 Jun 22;23:23. doi: 10.1186/s12991-024-00508-y (PMC11193281; doi:10.1186/s12991-024-00508-y)
Supplement: Supplementary file 1 — Supplementary Material 1 [file 12991_2024_508_MOESM1_ESM.docx]

Supplementary table 1. Risks of subsequent major mental disorders between the offspring of parents with or without migraine†.

|  | ASD | ADHD | Schizophrenia | Bipolar disorder | Depressive disorder |
| --- | --- | --- | --- | --- | --- |
| Offspring of parents without migraine (n, %) | 651 (0.3) | 3798 (1.7) | 653 (0.3) | 531 (0.2) | 3189 (1.4) |
| Offspring of parents with migraine (n, %) | 71 (0.3) | 541 (2.4) | 71 (0.3) | 81 (0.4) | 456 (2.0) |
| HR (95% CI) | 1.04 (0.82-1.34) | **1.37 (1.25-1.50)** | 1.00 (0.78-1.28) | **1.35 (1.06-1.71)** | **1.33 (1.21-1.47)** |

HR: hazard ratio; CI: confidence interval; ASD: autism spectrum disorder; ADHD: attention deficit hyperactivity disorder.

†: adjusting for demographic data and parental mental comorbidities.

**Bold** indicates statistical significance.
